# Effects of Modified Crohn’s Disease Exclusion Diet as Adjunctive Therapy on Clinical Remission and Nutrition in Pediatric Crohn’s Disease: A Real-World Study from China

**DOI:** 10.3390/children12111479

**Published:** 2025-11-02

**Authors:** Dongdan Li, Yan Kong, Xiaolin Ye, Tianzhuo Zhang, Feihong Yu, Jie Wu

**Affiliations:** 1 Division of Clinical Nutrition, Beijing Pediatric Hospital, Capital Medical University, National Center for Children’s Health, Beijing 100045, China; lidongdan1988@163.com; 2 Division of Gastroenterology, Beijing Children’s Medical Center, Capital Medical University, National Center for Children’s Health, Beijing 100045, Chinaopheliacheung@foxmail.com (T.Z.); yufeihong1@sina.com (F.Y.)

**Keywords:** Crohn’s disease, exclusive enteral nutrition, Crohn’s disease exclusion diet, children

## Abstract

**Highlights:**

**What are the main findings?**

• As an adjunct to infliximab therapy, Crohn’s Disease Exclusion Diet (CDED) demonstrated comparable efficacy to Exclusive Enteral Nutrition (EEN) in inducing disease remission in children with moderate-to-severe Crohn’s disease (CD) or mild CD with high-risk factors.
• CDED was more effective than EEN in improving physical growth (higher BMI Z-score) and reducing intestinal inflammation (lower fecal calprotectin) after 12 weeks of therapy.

**What is the implication of the main finding?**

• CDED may represent a practical adjunctive nutritional approach for Chinese children with CD receiving biologic therapy, particularly in settings where growth improvement and intestinal inflammation control are prioritized.
• These results provide preliminary support for considering CDED as a culturally adaptable dietary option in clinical nutrition practice for pediatric CD in China.

**Abstract:**

**Background/Objective:** Crohn’s disease exclusion diet (CDED) is used to induce remission and maintenance treatment of Crohn’s disease (CD), but its efficacy as adjuvant treatment of biological agents is not clear, especially for children with CD in China. The aim is to compare the synergistic induction of remission, as well as the effects on physical growth and nutritional indicators, of the CDED and Exclusive Enteral Nutrition (EEN), when used alongside infliximab as adjunctive therapies for children with CD. **Methods:** A retrospective analysis was conducted on newly diagnosed children with CD who were receiving infliximab treatment in combination with either CDED or EEN at the Department of Gastroenterology at Beijing Children’s Hospital between April 2022 and June 2025. The patients were divided into two groups: CDED and EEN. Changes in disease activity, physical growth indicators, nutritional status, and inflammatory markers were then compared between the two groups at six and twelve weeks post-treatment. **Results:** A total of 45 children with CD who met the inclusion and exclusion criteria were included in the study. Of these, 27 were boys (60%) and 18 were girls (40%), with an average age of (11.6 ± 2.9) years. Based on nutritional intervention, 19 patients were assigned to the CDED group and 26 to the EEN group. The clinical remission rates were 89.5% and 94.7% at 6 and 12 weeks post-treatment, respectively, in the CDED group and 88.5% and 84.6%, respectively, in the EEN group. At 12 weeks, the endoscopic remission rates were 47.1% (8/17) and 24.0% (5/26), respectively, in the CDED and EEN groups. There were no statistically significant differences between the two groups in terms of clinical remission or endoscopic remission (*p* > 0.05). Comparisons of physical growth indicators showed that, after 6 and 12 weeks of treatment, children in the CDED group had a significantly higher body mass index (BMI) for age Z-score than those in the EEN group (*p* < 0.05). Comparisons of serum nutritional and inflammatory markers revealed that, after 12 weeks of treatment, fecal calprotectin levels were significantly lower in the CDED group than in the EEN group (*p* < 0.05), with no significant differences observed in other markers. **Conclusions:** For children with moderate-to-severe or high-risk factors, CDED and EEN therapy as adjunctive treatment to infliximab demonstrate comparable efficacy in inducing disease remission. However, CDED was more effective than EEN at improving physical growth and reducing intestinal inflammation.

## 1. Introduction

Crohn’s disease (CD) is a chronic, recurrent inflammatory bowel disease. Over the past decade, there has been an upward trend in the incidence and prevalence of inflammatory bowel disease (IBD) in children worldwide. The annual incidence of CD in children in Asia has been reported to range from 0.3 to 15.3 per 100,000 [1]. Pediatric-onset CD is often more aggressive, extensive and severe, with a greater requirement for immunosuppressants and associated morbidity than CD with adult [2]. Despite significant progress in treating pediatric CD through the use of biologics, approximately one-third of children do not respond to initial anti-tumor necrosis factor alpha (anti-TNFα) therapy, and a further 30–40% experience treatment failure at a later stage [3].

Malnutrition is common in children with IBD, particularly CD. Studies from China suggest that the prevalence of malnutrition in children newly diagnosed with CD ranges from 49.6% to 63.6% [4,5]. Malnutrition can lead to changes in body composition, including muscle wasting and abnormal fat distribution, which are associated with poor outcomes in patients with CD [6]. Therefore, nutritional therapy plays a crucial role in the treatment of CD. One such therapy is exclusive enteral nutrition (EEN), which can effectively induce clinical remission, promote mucosal healing, and improve nutritional status and quality of life, and is recommended as first-line treatment for children with mild to moderate CD [7]. However, children with CD often have difficulty tolerating and adhering to EEN, which makes long-term use impractical. Some patients may experience a flare-up of inflammation when reintroducing foods after undergoing EEN induction therapy [8].

Diet plays a significant role in the pathogenesis of IBD [9]. Specialized dietary therapies have gradually become more prominent in the treatment of CD. The Crohn’s Disease Exclusion Diet (CDED) is a dietary therapy that combines partial enteral nutrition with specific food restrictions, aimed at reducing exposure to potential pro-inflammatory dietary components that may negatively impact the gut microbiota, immune response, and intestinal barrier function [10]. Studies have shown that the CDED is as effective as EEN in inducing clinical remission in children with mild to moderate CD. It is not only better tolerated and more compliant than EEN [11], but also promotes weight gain more effectively [12]. The latest ESPEN guidelines recommend CDED as an alternative treatment option to EEN for achieving disease remission in children with mild to moderate CD [13].

The value of enteral nutrition as an adjunctive therapy for patients with CD is increasingly recognized in clinical practice. A meta-analysis of CD in adults showed that enteral nutrition combined with infliximab was more effective than infliximab monotherapy at inducing and maintaining clinical remission [14]. Combining EEN with biologics may produce a synergistic effect, promoting disease remission more effectively. Current research on CDEDs primarily focuses on patients with uncomplicated CD without extraintestinal manifestations, patients with mild to moderate CD undergoing induction therapy, and patients eligible for biologic therapy as rescue treatment [15,16]. However, few studies have examined the use of CDED as an adjunctive therapy to infliximab. Furthermore, the applicability and efficacy of CDEDs based on Western dietary habits in Chinese children with CD have not been validated by clinical studies.

Therefore, this study aims to analyze the efficacy of CDED combined with infliximab in Chinese pediatric CD patients with moderate to severe active disease or high-risk factors, and to compare this with EEN as an adjunctive therapy. The study will provide evidence to inform the development of more suitable, personalized nutritional intervention schemes for Chinese children with CD.

## 2. Materials and Methods

### 2.1. Patients

A retrospective study was conducted at a tertiary children’s hospital. The study population consisted of children who were newly diagnosed with CD and admitted to the Department of Gastroenterology at Beijing Children’s Hospital, Capital Medical University, between April 2022 and June 2025. We collected retrospective data from the hospital’s electronic health records and endoscopy reporting system.

Inclusion criteria: (1) Newly diagnosed CD meeting the revised Porto criteria of the European Society for Pediatric Gastroenterology, Hepatology, and Nutrition (ESPGHAN) [17]; (2) Moderate to severe active disease, or mild active disease with high-risk factors (extensive intestinal involvement, intestinal strictures or perianal lesions); (3) Received infliximab combined with nutritional therapy: either EEN or CDED; (4) Age 0~18 years.

Exclusion criteria: (1) Concurrent gastrointestinal diseases such as intestinal tumors, intestinal tuberculosis, or chronic intestinal infections, or concomitant severe cardiovascular diseases, hematological malignancies, etc.; (2) Infliximab combined nutritional therapy for less than 12 weeks; (3) Children with overweight or obesity; (4) Incomplete case records.

**Figure 1:**
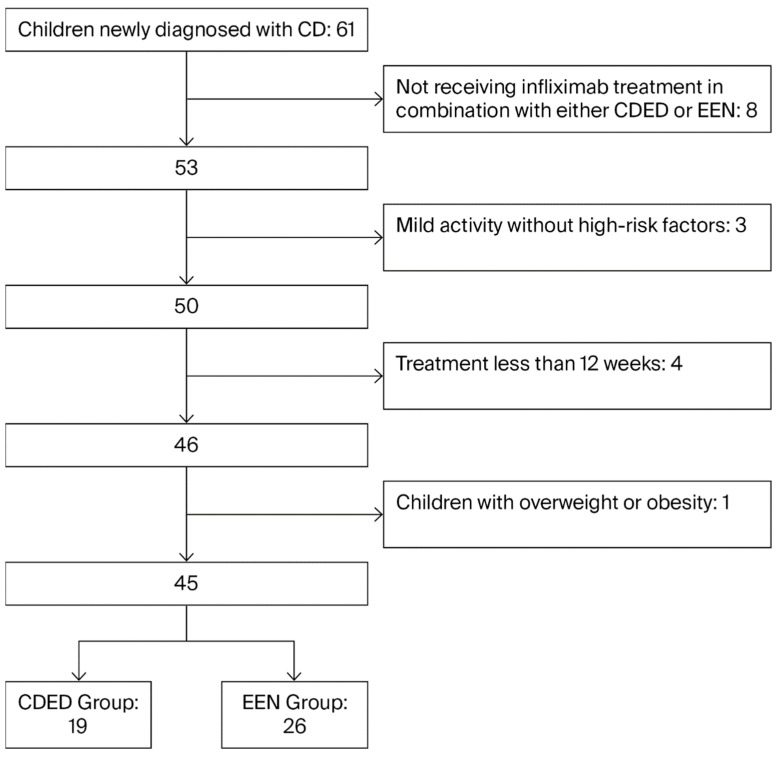
Inclusion and exclusion criteria.

Children were divided into the EEN group and the CDED therapy group based on their nutritional therapy regimens. The participant selection process was detailed in Figure 1.

### 2.2. Clinical Data Collection

Clinical data on children with CD was collected through the electronic medical record system. This included demographic information (gender and age), physical measurements (weight, height and body mass index), clinical manifestations (symptoms and disease activity), laboratory indicators (complete blood count, serum biochemistry, erythrocyte sedimentation rate and fecal calprotectin), endoscopy and treatment plans.

Clinical assessment: Based on the Montreal classification [18], the location of the disease is categorized as follows: L1 (terminal ileum), L2 (colon), L3 (ileum and colon), L4 (upper gastrointestinal tract). Disease behavior is categorized as follows: B1 (non-stenotic, non-penetrating), B2 (stenotic), B3 (penetrating), P-type (perianal lesions).

Clinical activity is assessed using the Pediatric Crohn’s Disease Activity Index (PCDAI) [19], which is categorized as either clinical remission (<10.0 points) or clinical activity (≥10.0 points).

Endoscopic activity is assessed using the simplified endoscopic score for Crohn’s disease (SES-CD) [20], and is categorized as endoscopic remission (≤2 points) or endoscopic activity (≥3 points).

The nutritional assessment indicators were calculated using the World Health Organization’s Anthro software [21]. The indicators were the height-for-age Z-score (HAZ) and the body mass index for age Z-score (BMIZ).

Nutritional therapy methods: (1) EEN nutritional therapy: This involves providing the child with total enteral nutrition using pediatric enteral nutrition formulas, which are administered either orally or via nasogastric feeding. No other food intake is permitted. (2) CDED dietary therapy: The first stage is a 6-week course (0~6 weeks), during which half of the patient’s diet consists of the CDED (prepared using traditional Chinese cooking methods), and the other half consists of partial enteral nutrition (PEN) therapy. The second stage is a 6-week course (7~12 weeks), during which patients can choose from a wider variety of foods, combined with 25% PEN therapy (see Table 1) [16,22]. The CDED and EEN regimen were implemented over 12 weeks as a home-based therapy. Patients and their families received intensive, face-to-face education and detailed materials from clinical dietitians prior to initiation. At the 6th and 12th weeks of treatment, the children were hospitalized for evaluation and received dietary guidance. Children who failed to return to the hospital on time or did not complete the 12-week nutritional therapy were excluded, thereby ensuring adherence.

The total energy requirements for patients in both groups were determined through a two-step process. Firstly, the resting metabolic energy (RME) was estimated using the Schofield equation, taking the child’s current body weight and height into account. The RME was then multiplied by activity stress adjustment factors to obtain the total energy requirement: ×1.3 well-nourished child at bed rest with mild-to-moderate stress; ×1.5 normally active child with mild-to-moderate stress, inactive child with severe stress; or child with minimal activity and malnutrition who requires catch-up growth; ×1.7 active child with severe stress; or active child who requires catch-up growth [23].

Efficacy evaluation: Comparison of disease activity and nutritional status between the CDED and EEN groups at weeks 6 and 12 of treatment. (1) Clinical disease activity and endoscopic scores: PCDAI, SES-CD scores; (2) Inflammatory markers: total white blood cells, platelets, C-reactive protein, erythrocyte sedimentation rate, fecal calprotectin; (3) Growth and development indicators: HAZ score, BMI Z score; (4) Serum nutritional indicators: serum total protein, albumin, prealbumin, triglycerides, total cholesterol, hemoglobin.

### 2.3. Statistical Analysis

All continuous variables were tested for normality and homogeneity of variance. The statistical methods were selected based on the data distribution and study design. For normally distributed data, the paired *t*-test was used for within-group comparisons, while the independent samples *t*-test was applied for comparisons between different groups. Non-normally distributed data were analyzed using the Wilcoxon signed-rank test for paired samples and the Mann–Whitney U test for independent groups. Categorical data, presented as numbers (percentages), were compared using the Chi-square test or Fisher’s exact test. A *p*-value of less than 0.05 was defined as statistically significant. All analyses were performed with SPSS Statistics version 22.0.

## 3. Results

### 3.1. Study Population and Baseline Characteristics

A retrospective review of medical records identified 61 children who were newly diagnosed with CD at our center between April 2022 and June 2025. After applying the inclusion and exclusion criteria, a final cohort of 45 patients was included for analysis. including 27 boys (60%) and 18 girls (40%), with an average age of 11.6 years (±2.9). Of these children, 26 had moderate to severe disease activity and 19 had mild disease activity combined with high-risk factors (extensive intestinal involvement, intestinal strictures or perianal lesions). These patients were categorized into two groups based on the nutritional therapy they received alongside infliximab: the CDED group (n = 19) and the EEN group (n = 26). A comparison of the two groups’ baseline data, including gender, age, initial disease activity, affected sites, disease behavior, fecal calprotectin, SES-CD scores, and nutritional status (BMIZ and HAZ), showed no statistically significant differences (*p* > 0.05) (see Table 2).

### 3.2. Comparison of Clinical and Endoscopic Activity Between the Two Groups of Children After Treatment

Six weeks after treatment, the clinical remission rate was 89.5% (17/19) in the CDED group and 88.5% (23/26) in the EEN group. At 12 weeks, the remission rates were 94.7% (18/19) and 84.6% (22/26). There were no statistically significant differences between the two groups in terms of clinical remission rates or PCDAI scores (*p* > 0.05). Following 12 weeks of treatment, 17 patients in the CDED group underwent endoscopic follow-up, achieving an endoscopic remission rate of 47.1% (8/17). In the EEN group, 25 patients underwent endoscopic follow-up, achieving an endoscopic remission rate of 24.0% (6/25). There were no statistically significant differences in endoscopic remission rates or SES-CD scores between the two groups (*p* > 0.05) (see Table 3).

### 3.3. Comparison of Physical Growth Indicators Between the Two Groups of Children After Treatment

Intragroup analysis showed that after six weeks of treatment, children in the CDED group had a statistically significant improvement in BMI Z-scores compared to baseline (*p* = 0.001), whereas the improvement in the EEN group did not reach statistical significance (*p* > 0.05); neither group exhibited significant changes in HAZ scores relative to baseline (*p* > 0.05). At 12 weeks of treatment, both groups demonstrated significant improvements in BMI Z-scores compared to baseline (*p* = 0.034 and *p* = 0.014), while HAZ scores remained stable without significant changes (*p* > 0.05) (Table 4).

A comparison of physical growth indicators between the two groups of patients after treatment showed that the BMI Z scores of patients in the CDED group were significantly higher than those in the EEN group at 6 weeks and 12 weeks (*p* = 0.039, *p* = 0.03), while there was no statistically significant difference in HAZ scores between the two groups at 6 weeks and 12 weeks (*p* > 0.05) (see Table 5).

### 3.4. Comparison of Serum Nutritional and Inflammatory Markers Between the Two Groups of Children After Treatment

A comparison of serum nutritional markers between the two groups of patients after treatment revealed that there were no statistically significant differences in serum albumin, total protein, prealbumin, triglycerides, total cholesterol, and hemoglobin levels between the two groups at 6 weeks and 12 weeks (*p* > 0.05).

Comparing inflammatory markers between the two groups of patients after treatment, the results showed that there were no statistically significant differences in white blood cell count, platelet count, C-reactive protein, and erythrocyte sedimentation rate at 6 weeks (*p* > 0.05); at 12 weeks, fecal calprotectin levels were significantly higher in the EEN group than in the CDED group (*p* = 0.007), with no other statistically significant differences (see Table 6).

## 4. Discussion

Anti-tumor necrosis factor (TNF) biologics enable rapid onset of action and induce disease remission, representing a major advancement in the treatment of CD. TNF inhibitors have been shown to be highly effective in inducing and maintaining steroid-free clinical remission, promoting mucosal healing and reducing the risk of surgical and intestinal stricture complications. This has a positive impact on the natural course of CD [24]. However, with the widespread use of anti-TNF drugs, an increasing number of patients are experiencing secondary non-response or resistance. A population-based study revealed that among 82.5% of children with CD treated with anti-TNF agents, 30.7% did not respond within 1 year, and 61.9% experienced treatment failure within 5 years [25]. Against this backdrop, exploring adjunctive therapeutic strategies that enhance drug efficacy and delay the onset of resistance has become particularly important. Although nutritional therapies, such as EEN, have traditionally been primarily used for inducing remission in children with mild to moderate CD, further exploration is needed to determine whether specialized diets and nutritional therapies can serve as adjunctive treatment options to infliximab, synergistically promoting and maintaining remission in CD patients to some extent, while also holding potential for improving nutritional status and long-term prognosis.

Previous studies have shown that the clinical remission rate in children with CD treated with infliximab for 10 weeks ranged from 58.9% to 85% [26]. A study in adult CD patients demonstrated that EEN combined with biologic therapy enhances the induction of remission compared to monotherapy with biologics, aids in the recovery of body composition, optimizes the pharmacokinetics of biologics, and reduces inflammatory cytokine levels [27]. In this study, the clinical remission rate after 12 weeks of CDED combined with TNF therapy reached 94.7%, the remission rate for EEN combined with TNF therapy was 84.6%, there was no statistically significant difference between the two rates. The remission rates in both groups confirm that CDED and EEN were equally effective when used alongside infliximab to induce clinical remission in children with Crohn’s disease. Additionally, all children in this study were either moderately to severely active or had high-risk factors for CD. A pediatric real-world cohort study showed that CDED combined with PEN as an adjunctive therapy for refractory severe patients is effective, capable of effectively inducing remission in clinically severe patients and those with isolated colonic phenotypes [28].

Endoscopic mucosal healing has become the primary therapeutic goal and endpoint for CD, with significance extending far beyond symptom control. It correlates with long-term clinical remission and quality of life in pediatric patients [29]. This study assessed and compared endoscopic mucosal healing outcomes. At 12 weeks, the endoscopic remission rate for CDED combined with TNF was 47.1%, compared to 24.0% for EEN combined with TNF, a difference that was not statistically significant (*p* = 0.120). Previous studies have shown that the endoscopic mucosal healing rate ranges from 22% to 42% during the 3~12 months following initiation of anti-TNFα therapy [3]. Although the clinical remission rate and endoscopic remission rate were higher in the CDED plus infliximab group than in the EEN plus infliximab group in this study, the difference between the two groups did not reach statistical significance. Meanwhile, compared with the six-week mark, the clinical remission rate decreased in the EEN plus infliximab group at 12 weeks. In contrast, the clinical remission rate increased in the CDED plus infliximab group, though this difference was not statistically significant. Further research involving larger sample sizes is required to definitively establish whether there are significant differences in the long-term sustainability of remission between these two effective nutritional strategies. Additionally, at 12 weeks, the fecal calprotectin levels in the CDED plus infliximab group were significantly lower than those in the EEN plus infliximab group (*p* = 0.007), with both groups starting from comparable baseline levels (*p* = 0.797). This significant reduction suggests that the CDED regimen may be more effective in reducing intestinal inflammation than the EEN regimen. A previous study by Levine et al. in patients with CD also demonstrated that at week 12 of treatment, CDED combined with PEN significantly outperformed EEN in inducing sustained remission. It further indicated that although fecal calprotectin levels decreased significantly from baseline in both groups by week 6 with no statistically significant difference between them, the CDED + PEN group showed a sustained downward trend in this marker between weeks 6 and 12, whereas the EEN group exhibited a rebound [16]. Additionally, studies have shown that for CD children with significant clinical remission but elevated fecal calprotectin levels, CDED + PEN can serve as an effective adjunctive treatment to drug therapy [30].

Dietary and nutritional therapy, as an integral component of comprehensive treatment for CD, has consistently been a focal point of clinical attention due to its direct impact on children’s nutritional status. The effects of different nutritional intervention protocols on children’s growth indicators and nutritional parameters can provide evidence for clinical decision-making. This study showed that patients receiving CDED + PEN had significantly higher BMI-Z scores at 6 and 12 weeks of treatment than EEN patients. This suggests that adjunctive CDED therapy may be more effective than EEN at promoting weight gain in children. Previous studies on children with mild-to-moderate CD have also indicated that CDED combined with PEN is comparable to EEN in inducing remission, but there may be differences in improving nutritional indicators. A study indicated that EEN and CDED show no significant difference in improving nutrition [16]. However, a real-world study showed that compared with the EEN group, CDED + PEN treatment resulted in significant increases in weight gain and BMI-Z scores [12]. Another recent pediatric multicenter randomized controlled trial also demonstrated that compared with EEN, CDED treatment significantly improved BMI-Z scores in CD patients [31]. Additionally, studies have shown that, compared to the Mediterranean diet, CDED significantly reduces BMI while improving body composition, with reduced fat mass and increased fat-free mass at 12 weeks, with these effects sustained up to 24 weeks [32]. Our study also supports the potential advantage of CDED in improving children’s nutritional status (BMI growth), particularly for those requiring balanced growth and development alongside malnutrition management, thereby achieving disease control while optimizing nutrition.

The CDED dietary plan was initially developed by an Israeli research team and subsequently validated and promoted in several Western countries [33]. Most studies have been conducted within the context of Western dietary cultures, and there is still limited evidence regarding its application in Asian populations, particularly among Chinese children. A localized CDED plan offers multiple advantages, such as greater cost-effectiveness, better compliance (aligning with daily dietary habits), and stronger sustainability (aiding in the establishment of healthy dietary patterns). This study is the first systematic evaluation of the efficacy of a CDED protocol modified based on traditional Chinese dietary culture (using traditional Chinese cooking methods, adhering to Chinese dietary customs, and aligning with Chinese children’s taste preferences and nutritional needs) in Chinese children with CD. The results showed that, for Chinese children with CD in moderate to severe active disease or with high-risk factors, the modified CDED regimen was comparable to EEN in terms of improving disease activity as an adjunctive therapy, while demonstrating significant in promoting physical development and reducing intestinal inflammation. This suggests that a CDED regimen appropriately modified for local conditions offers additional clinical benefits beyond disease control, providing a superior option for nutritional therapy in Chinese children with CD.

This study has certain limitations. Firstly, it is a retrospective study with a small sample size, which may introduce bias. Further validation is needed through prospective studies with larger sample sizes. Secondly, the assessment of physical growth and nutritional status is incomplete, lacking indicators such as bone density, body composition analysis and vitamin levels. Thirdly, the observation period was short, failing to adequately assess the long-term efficacy of nutritional supplementation and its impact on growth and development. Finally, only a very small number of children with CD receiving infliximab monotherapy during the study period, resulting in the lack of comparison between infliximab plus nutritional intervention and infliximab monotherapy. Therefore, future prospective studies with an appropriately sized control group are required to directly evaluate the incremental benefit of nutritional therapy in combination with biologic treatment.

## 5. Conclusions

In summary, for children with moderate-to-severe CD or those with high-risk factors, CDED was as effective as EEN in inducing both clinical and endoscopic remission when used as an adjunctive therapy to infliximab. However, CDED had superior advantages in terms of improving physical growth (as indicated by higher BMI Z-scores) and reducing intestinal inflammation (as shown by lower fecal calprotectin levels). These findings suggest that, for Chinese children with CD receiving biologic therapy, CDED may represent a practical nutritional approach, particularly in clinical settings where growth improvement and inflammation control are prioritized.

## Figures and Tables

**Table 1:** CDED food list.

	Phase 1 (0~6 Weeks)	Phase 2 (7~12 Weeks)
	50%PEN + modified diet	25%PEN + modified diet
Mandatory Foods	A total of 150–200 g of fresh chicken breast, 2 eggs, 2 bananas, 1 apple (peeled), 2 medium-sized fresh potatoes (peeled, boiled, and cooled).	Same as Phase I
Allowed Foods	Replace chicken breast with fresh, lean white fish (1 serving, up to 200 g, once a week,); white rice, rice noodles, rice flour (made from pure rice ground into flour, unlimited); 1 avocado; 5 strawberries; 1 slice of melon (Hami melon or white melon); 2 tomatoes; 2 cucumbers (peeled); 1 carrot; 1 cup of raw spinach; 3 lettuce leaves.	Replace chicken breast with fresh, lean white fish once a week (maximum 200 g per serving); one can of tuna per week (only in olive oil or low-erucic-acid canola oil); one serving of fresh, unprocessed lean beef steak per week (maximum 200 g); white rice, rice noodles, rice flour (made solely from ground rice, unlimited); ½ sweet potato (as a substitute for regular potatoes, once daily); 1 slice of whole-grain bread daily (homemade, yeast-free); ½ cup of dried beans daily (not canned or frozen); quinoa—unlimited; ½ cup of dry oatmeal per week; 1 avocado; 5 strawberries or 10 blueberries; 1 pear, peach, or kiwi; 1 slice of melon (Hami melon or white melon); 2 tomatoes; 2 cucumbers (peeled); 1 carrot; 1 cup raw spinach; 3 lettuce leaves; 1 zucchini or 4–6 fresh mushrooms or 2 broccoli florets or cauliflower florets. Starting from week 10, other fruits and vegetables can be gradually introduced into the diet. High-fiber fruits can be consumed in small amounts, such as ½ cup mango, pineapple, or orange slices. At this stage, all vegetables can be included in the diet except kale, leeks, asparagus, artichokes, and celery.
Seasoning	Oil: Olive oil, low-erucic-acid canola oil. Spices (pure): salt, pepper powder, cinnamon, cumin, turmeric. Fresh herbs: mint, oregano, cilantro, rosemary, sage, basil, thyme, dill, parsley. Other seasonings: onion, garlic, ginger, fresh lemon juice.	Same as Phase I
Excluded foods	Foods not included in the list of permitted or mandatory foods are not allowed to be consumed. If a food is not included in the list of permitted foods, patients are required to treat it as a prohibited food.	Foods not on the list should be avoided.
Cooking method	Adopt Chinese-style cooking methods, such as stir-frying, steaming, boiling, and stewing, and avoid using seasonings containing preservatives or additives, such as soy sauce and cooking wine.	Same as Phase I

**Table 2:** Comparison of baseline data on general and clinical conditions between the two groups of children with CD.

Indicator	EEN (N = 26)	CDED (N = 19)	*t*/*χ*^2^/*Z*	*p*
Gender, male [*n* (*%*)]	14	13	0.972	0.324
Age [*M* (*P*_25_, *P*_75_), year]	12.0 (10.8, 13.0)	12.0 (9.0, 14.0)	−0.082	0.935
PCDAI [*M* (*P*_25_, *P*_75_)]	31.3 (17.5, 48.8)	32.5 (20.0, 37.5)	−0.484	0.629
SES-CD [*x* ± *s*]	14.4 ± 6.0	15.0 ± 5.8	−0.344	0.732
BMIZ [*M* (*P*_25_, *P*_75_)]	−1.3 (−2.9, −0.8)	−1.2 (−2.4, 0.1)	−1.092	0.275
HAZ [*M* (*P*_25_, *P*_75_)]	0.1 (−0.9, 0.9)	0.3 (−0.6, 1.3)	−1.034	0.301
FC [*M* (*P*_25_, *P*_75_)]	1800 (1800, 1800)	1800 (1800, 1800)	−0.257	0.797
Site of lesion [*n* (%)]				
Terminal ileum [*n* (%)]	16	12	0.012	0.912
Colon [*n* (%)]	15	9	0.470	0.493
Ileum and colon [*n* (%)]	8	8	0.616	0.433
Upper gastrointestinal tract [*n* (%)]	13	4	-	0.065
Intestinal stricture [*n* (%)]	9	3	-	0.191
Perianal lesions [*n* (%)]	8	6	0.003	0.954

Note: CDED: Crohn’s Disease Exclusion Diet; EEN: Exclusive Enteral Nutrition; PCDAI: Pediatric Crohn’s Disease Activity Index; SES-CD: Simple Endoscopic Score for Crohn’s Disease; BMI Z: Body Mass Index for age z-score; HAZ: Height-for-Age z-score; FC: Fecal Calprotectin.

**Table 3:** Comparison of PCDAI scores and SES-CD scores between the two groups of children after treatment.

Indicator	6 Weeks After Treatment		*χ*^2^/*Z*	*p*	12 Weeks After Treatment		*χ*^2^/*Z*	*p*
	CDED	EEN			CDED	EEN		
PCDAI [*M* (*P*_25_, *P*_75_)]	0 (0, 5)	0 (0, 5)	−0.039	0.969	0 (0, 2.5)	0 (0, 5)	−0.364	0.716
Clinical remission rate [*n* (%)]	17 (89.5%)	23 (88.5%)	-	1.000	18 (94.7%)	22 (84.6%)	-	0.378
SES-CD [*M* (*P*_25_, *P*_75_)]	-	-	-	-	3 (1.5, 6)	5 (2.5, 9)	−1.419	0.156
Endoscopic remission rate [n (%)]	**-**	-	-	-	8 (47.1%)	6 (24.0%)	2.421	0.120

Note: CDED: Crohn’s Disease Exclusion Diet; EEN: Exclusive Enteral Nutrition; PCDAI: Pediatric Crohn’s Disease Activity Index; SES-CD: Simple Endoscopic Score for Crohn’s Disease.

**Table 4:** Changes in BMI z-scores and HAZ scores before treatment and 6 weeks and 12 weeks after treatment in two groups of children.

Indicator	BMI Z [*x* ± *s*] [*M* (*P*_25_, *P*_75_)]							HAZ [*x* ± *s*] [*M* (*P*_25_, *P*_75_)]						
	Baseline	6 Weeks After Treatment	t/Z	*p*	12 Weeks After Treatment	t/Z	*p*	Baseline	6 Weeks After Treatment	t/Z	*p*	12 Weeks After Treatment	t/Z	*p*
CDED	−1.1 ± 1.7	−0.3 ± 1.3	−3.950	0.001	−0.0 ± 1.3	−2.290	0.034	0.4 ± 1.2	0.3 ± 1.2	1.022	0.320	0.3 ± 1.1	0.710	0.487
EEN	−1.3 (−2.9, −0.8)	−1.1 (−1.9, −0.6)	−1.880	0.060	−0.9 (−1.6, 0.0)	−2.462	0.014	0.1 (−0.9, 0.9)	0.0 (−0.8, 0.7)	−0.606	0.545	0.2 (−0.8, 0.6)	−0.495	0.620

Note: CDED: Crohn’s Disease Exclusion Diet; EEN: Exclusive Enteral Nutrition; BMI Z: Body Mass Index for age z-score; HAZ: Height-for-Age z-score.

**Table 5:** Changes in BMI z-scores and HAZ scores at 6 weeks and 12 weeks post-treatment in the two groups of children.

Group	BMIZ [*x* ± *s*] [*M* (*P*_25_, *P*_75_ )]		HAZ [*x* ± *s*]	
	6 Weeks After Treatment	12 Weeks After Treatment	6 Weeks After Treatment	12 Weeks After Treatment
CDED	−0.4 (−1.3, 0.6)	−0.0 ± 1.3	0.3 ± 1.2	0.3 ± 1.1
EEN	−1.1 (−1.9, −0.6)	−0.9 ± 1.4	−0.1 ± 1.4	−0.1 ± 1.4
*t*/*Z*	−2.068	−2.251	−1.059	−1.115
*p*	0.039	0.030	0.296	0.271

Note: CDED: Crohn’s Disease Exclusion Diet; EEN: Exclusive Enteral Nutrition; BMI: Body Mass Index for age z-score; HAZ: Height-for-Age z-score.

**Table 6:** Comparison of serum nutritional and inflammatory markers between the two groups at baseline and 6 weeks and 12 weeks post-treatment.

Indicator	Baseline		*t*/*Z*	*p*	6 Weeks After Treatment		*t*/*Z*	*p*	12 Weeks After Treatment		*t*/*Z*	*p*
	EEN	CDED			CDED	EEN			CDED	EEN		
TP [x ± s, g/L]	66.8 ± 6.0	70.5 ± 4.7	2.227	0.031	74.4 ± 3.8	72.2 ± 7.4	−1.058	0.298	74.4 ± 3.6	71.6 ± 6.1	−1.888	0.066
ALB [*M* (*P*_25_, *P*_75_), g/L]	35.0 ± 4.5	35.7 ± 4.4	0.509	0.613	42.5 (40.6, 44.4)	43.9 (41.8, 45.3)	−0.865	0.387	43.9 (43.0, 45.8)	43.8 (42.1, 45.2)	−0.813	0.416
PA [*x* ± *s*, g/L]	102.5 (78.3, 141.5)	117.0 (90.0, 116.0)	−1.460	0.144	223.8 ± 53.7	221.9 ± 48.9	−0.537	0.597	201.6 ± 43.8	200.2 ± 56.3	−0.088	0.931
TG [*x* ± *s*, mmol/L]	0.9 ± 0.3	1.1 ± 0.5	1.270	0.211	1.4 ± 0.7	1.0 ± 0.4	−1.757	0.093	0.9 ± 0.2	0.9 ± 0.5	0.732	0.469
TC [*M* (*P*_25_, *P*_75_), mmol/L]	3.3 ± 0.5	3.6 ± 0.9	1.269	0.216	3.9 (3.5, 4.3)	3.5 (3.2, 3.7)	−1.950	0.051	3.4 ± 0.6	3.4 ± 0.4	0.349	0.728
HB [*x* ± *s*, g/L]	103.5 (98.3, 111.3)	114.0 (104.0, 123.0)	−2.267	0.023	126.9 ± 10.1	120.1 ± 12.0	−1.855	0.072	130 (120, 134)	125.0 (116.5, 136.3)	1.855	0.072
WBC [*M* (*P*_25_, *P*_75_), ×10^9^/L]	7.9 (6.4, 10.1)	10.1 (7.3, 14.1)	−1.701	0.089	6.3 (5.0, 9.0)	5.5 (4.3, 6.7)	−1.770	0.077	6.62 (5.4, 8.2)	5.5 (4.4, 7.9)	−1.023	0.306
PLT [*x* ± *s*, ×10^12^/L]	419.5 ± 118.7	441.8 ± 123.4	0.613	0.543	328.2 ± 88.5	310.3 ± 91.3	−0.603	0.550	291.5 ± 86.2	314.2 ± 123.1	0.688	0.495
CRP [*M* (*P*_25_, *P*_75_), mg/L]	28.0 (13.8, 48.3)	11.0 (0.5, 31.0)	−1.923	0.054	10 (5, 10)	10 (4, 10)	−0.055	0.956	10 (10, 10)	10 (10, 10)	−0.016	0.987
ESR [*M* (*P*_25_, *P*_75_), mm/h]	24.0 (7.8, 32.5)	34.0 (17.0, 48.0)	−1.541	0.123	7 (5, 17.5)	6 (3, 10)	−1.149	0.250	11.5 ± 7.5	10.1 ± 11.0	−0.482	0.632
FC [*M* (*P*_25_, *P*_75_), ug/g]	1800 (1800, 1800)	1800 (1800, 1800)	−0.257	0.797	-	-	-	-	60 (30, 524)	924.5 (126.3, 1800)	−2.712	0.007

Note: CDED: Crohn’s Disease Exclusion Diet; EEN: Exclusive Enteral Nutrition; TP: Total Protein; ALB: Albumin; PA: Prealbumin; TG: Triglycerides; TC: Total Cholesterol; HB: Hemoglobin; WBC: White Blood Cell; PLT: Platelet Count; CRP: C Reactive Protein; ESR: Erythrocyte Sedimentation Rate; FC: Fecal Calprotectin.

## Data Availability

The original contributions presented in the study are included in the article, further inquiries can be directed to the corresponding author.

## Abbreviations

CD	Crohn’s Disease
EEN	Exclusive Enteral Nutrition
CDED	Crohn’s Disease Exclusion Diet
PEN	Partial Enteral Nutrition
IBD	Inflammatory Bowel Disease
BMI	Body Mass Index
HAZ	Height-for-age Z-score
SES-CD	Simplified Endoscopic Score for Crohn’s Disease
PCDAI	Pediatric Crohn’s Disease Activity Index
ESPGHAN	European Society for Pediatric Gastroenterology, Hepatology, and Nutrition
TP	Total Protein
ALB	Albumin
PA	Prealbumin
TG	Triglycerides
TC	Total Cholesterol
HB	Hemoglobin
WBC	White Blood Cell
PLT	Platelet Count
CRP	C Reactive Protein
ESR	Erythrocyte Sedimentation Rate
FC	Fecal Calprotectin
TNF	Anti-tumor Necrosis Factor
RME	Resting Metabolic Energy
